# Validation of Gait Kinematics With Ramp and Stair Ascent and Descent Revealed by Markerless Motion Capture in Simulated Living Space: Test-Retest Reliability Study

**DOI:** 10.2196/66886

**Published:** 2025-05-15

**Authors:** Daiki Shimotori, Kenji Kato, Tatsuya Yoshimi, Izumi Kondo

**Affiliations:** 1 Laboratory of Practical Technology in Community, National Center for Geriatrics and Gerontology, Obu, Japan; 2 Laboratory of Clinical Evaluation with Robotics, Assistive Robot Center, National Center for Geriatrics and Gerontology, 7-430, Morioka-cho, Obu, 474-8511, Japan, 81 562-46-2311, 81 562-48-2373; 3 Assistive Robot Center, National Center for Geriatrics and Gerontology, Obu, Japan

**Keywords:** markerless motion capture, living laboratory, level walking, ramp walking, stair climbing, kinematics, measurement error, absolute reliability, gait, walk, stair, ramp, rehabilitation, reliability, simulated living space, simulation, motion capture, gait kinematics

## Abstract

**Background:**

In recent years, there has been an increasing demand for markerless motion capture systems, which are being widely used in biomechanical and clinical research. Furthermore, by using a markerless motion capture system in a laboratory environment that mimics living spaces, the data acquired on various activities of daily living, such as level walking, ramp walking, and stair ascent and descent, should more closely resemble that of real-life activities. However, the absolute reliability of gait parameters in this context is still unclear.

**Objective:**

The aim of this study was to evaluate the reliability of a markerless motion capture system in assessing the ascent and descent of ramps and stairs during walking in a simulated living space.

**Methods:**

A total of 21 healthy participants performed level walking, ramp and stair ascent and descent on two separate days, with at least a 24-hour interval between sessions. Joint angles were measured using 27 synchronized cameras with a markerless motion capture application, Theia3D (Theia Markerless Inc), and analyzed in Visual3d for all planes of motion at the hip-, knee-, and ankle-joints. The absolute reliability of day-to-day reproducibility was assessed using full-curve analysis (root mean square difference [RMSD]) and discrete point analysis of gait events using the standard error of measurement (SEM). SEM was calculated only for level walking and ramp ascent and descent, where gait events were correctly detected.

**Results:**

The SEM values for level walking and ramp ascent and descent were all below the 5-degree threshold. However, while RMSD values were generally below 5°, this threshold was exceeded for knee-joint flexion-extension angles during ramp ascent and stair ascent (5.07° and 5.64°, respectively).

**Conclusions:**

The markerless motion capture system in the living laboratory setting demonstrated a high degree of accuracy for various environments and gait types. The low SEM values obtained indicate good reliability for joint angle measurements across different days. The slightly higher RMSD values for knee-joint angles during ramp and stair ascent may reflect the system’s ability to capture the adaptations in joint kinematics in response to changes in gait conditions. These measurements in a living laboratory environment validated the absolute reliability of various gait parameters not only in level walking but also in ramp and stair ascent and descent. The findings suggest potential clinical applications and research opportunities, including the development of assistive devices and robots, using markerless motion capture in more natural living situations, rather than in controlled environments.

## Introduction

Gait serves as an indicator of an individual’s health status and physical function [1]. In particular, gait speed has been widely used as a general assessment parameter due to its simple measurement [2], and issue with gait speed have been associated with cognitive decline [3], survival rates [4], and body-related quality of life [5]. However, gait is a complex biomechanical process that requires comprehensive assessment of cadence, rhythm, variability, and asymmetry [6]. Furthermore, incorporating biomechanical indices, particularly joint angles, enables identification of specific gait impairments that may not be detected by conventional parameters alone. A detailed analysis of this kind is essential for optimizing rehabilitation and robot-assisted interventions as it allows strategies to be developed that are tailored to individual movement patterns and pathological conditions.

To gain further insight into gait mechanics, data from 3D motion capture systems that provide joint angles have been used. This information allows the identification of specific joint angles that contribute to gait abnormalities, which can then be targeted for interventions by way of rehabilitation, assistive devices, and robotics. For example, Anang et al [7] used 3D motion analysis to identify a characteristic joint angle pattern in patients with chronic stroke, specifically a reduced knee-joint flexion during the swing phase of walking. They suggested that this finding could be a useful indicator for targeted interventions aimed at improving overall gait function in stroke survivors. Similarly, Schmitt et al [8] used 3D motion analysis to investigate the effects of knee osteoarthritis on lower extremity joint mechanics during walking. They found that patients with knee osteoarthritis exhibited reduced hip-joint extension and increased ankle dorsiflexion compared with healthy controls. These alterations in hip- and ankle-joint angles were attributed to compensatory mechanisms adopted by patients to reduce knee-joint loading and pain. These studies highlight the importance of considering the entire lower extremity when assessing the impact of disease on gait and when planning interventions. Furthermore, joint angle data can inform the design and development of assistive devices and robots. For instance, exoskeletons and powered orthoses can provide assistive torque at specific joint angles to support and enhance desired motions [9]. Similarly, robot-assisted treadmill training can guide patients through specific joint angle trajectories that promote optimal gait patterns [10].

Despite the benefits of kinematic data, traditional marker-based 3D motion capture systems present several challenges. These systems generally require subjects to wear specific attire and footwear, and the process of conducting full-body measurements can be time-consuming. In addition, markers must be attached to anatomical landmarks for accurate measurements [11]. In some cases, it is difficult to attach markers to landmarks while wearing orthotics or using robots, which may compromise the validity of the gait data collected. Under such constraints, subjects are often asked to walk as usual, but this may not accurately represent their natural gait patterns. Consequently, the validity of gait data collected using marker-based 3D motion capture systems has been questioned [11].

Recently, significant advancements in artificial intelligence have facilitated the development of markerless motion capture technology [12]. This technology offers a convenient method of measurement without the requirement to remove clothing or shoes, making it a promising option for integration in clinical [13] and sports settings [14]. However, although this simplifies the process of taking measurements, it should not be at the cost of accuracy, and the interpretation of data must be carefully judged. Among the many markerless motion capture systems, which include Microsoft Kinect [15], OpenPose (developed by Carnegie Mellon University) [16], and DeepLabCut (developed by Harvard University) [17], Theia3D (developed by Theia Markerless Inc) has achieved practical application in a wide range of clinical and sports research, as well as in the production of animations. This is because of its demonstrated accuracy and reproducibility in measuring level walking, as validated by several studies comparing its performance against traditional marker-based motion capture systems [18,19]. Therefore, it is expected that Theia3D can also be applied to various activities of daily living (ADLs) besides level walking, which has been the main target of measurements to date. However, how accurate measurements of such ADLs might be remains unclear.

Kato et al [20] have developed a “living laboratory“ to facilitate ADLs assessment with Theia3D and to study the impact of assistive robots on ADLs in a simulated living environment. This living laboratory includes an indoor environment as well as a simulated outdoor environment to mimic natural living conditions. However, the reliability of markerless motion capture in the living laboratory setting has not been investigated in detail. Among the various ADLs studied previously, ramp and stair ascent and descent pose a higher risk of falls than level-ground walking [21,22], and ramp walking poses an even higher risk of falls compared with stair climbing [23]. These tasks require complex postural control and adaptations to environmental conditions, such as inclination and step height, making them particularly challenging for fall prevention [24]. Stair climbing was one of the more demanding tasks, as it required near-maximal muscle strength and a greater range of joint motion [25,26]; accordingly, it is included in the Barthel Index for the assessment of ADLs in older adults [27]. Therefore, it is clinically important to investigate the application and reliability of Theia3D for these activities. While previous studies have validated Theia3D for level walking, its reliability in assessing more complex movements in a simulated living environment remains unclear due to the increased variability and dynamic adaptations required for tasks such as stair and ramp walking. For gait analysis, reliability must be evaluated based both on overall movement patterns throughout the gait cycle and specific gait events, as these reflect both kinematic consistency and the precision of localized measurements [28]. Establishing its reliability for such movements is essential not only for research applications but also for clinical assessments and assistive technology development. Therefore, the purpose of this study was to evaluate the reliability of Theia3D in assessing the ascent and descent of ramps and stairs using the method of Riazati et al [29] to calculate between-day repeatability and representative values during walking.

## Methods

### Design of the Living Laboratory

**Figure 1.**
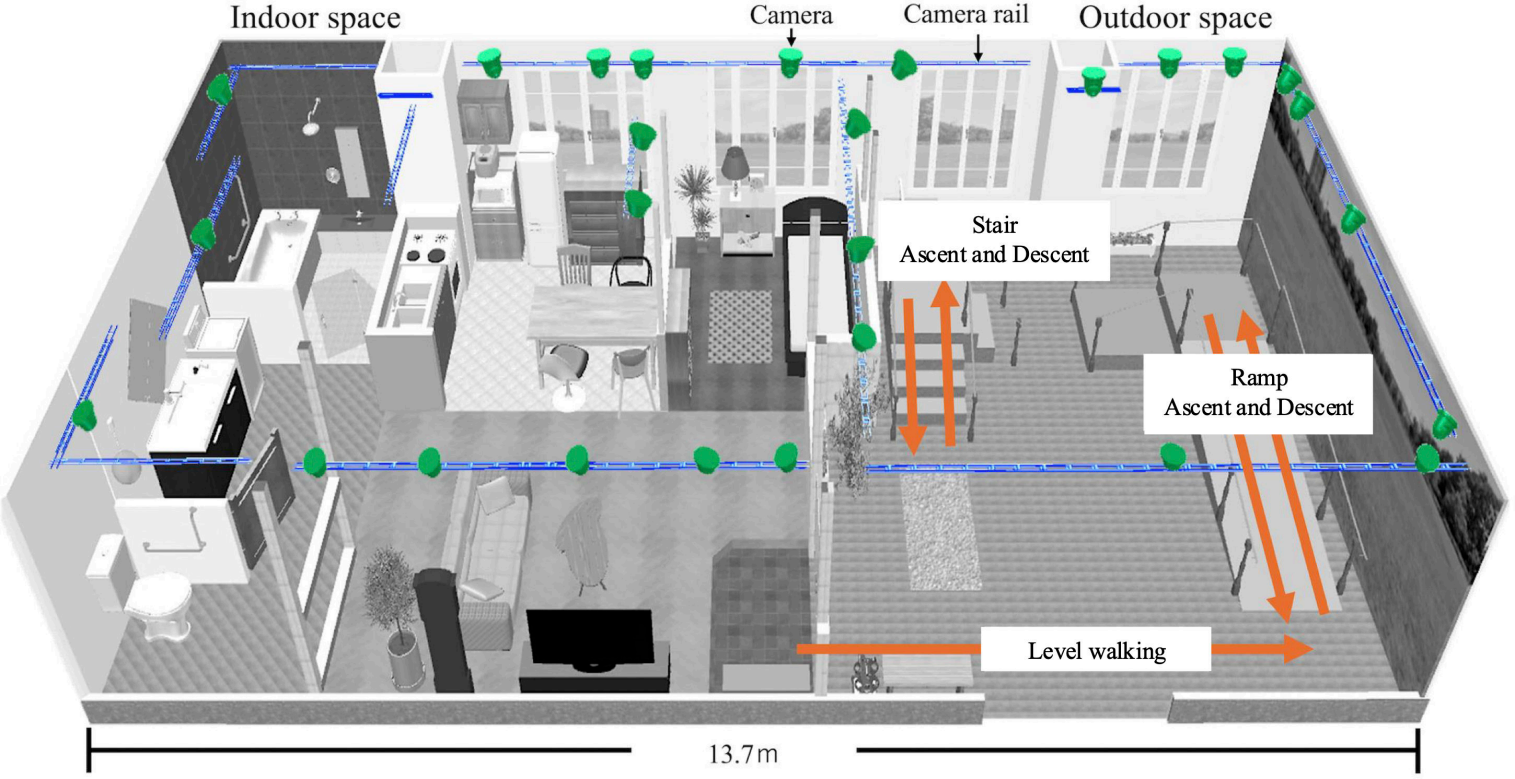
The living laboratory layout showing the positions of 27 cameras for markerless motion capture.

The living laboratory (Figure 1) was designed to simulate outdoor, home indoor, and nursing home environments [20]. It includes an indoor living space with a bedroom, living room, dining room, kitchen, washroom, bathroom, toilet, and entrance, as well as outdoor spaces including rough terrain, stairs, and ramps equipped with removable handrails. The stairs and ramp configurations were designed in accordance with the Building Standards Act of Japan and its Enforcement Ordinance [30]. The gradient of the 3.25-m-long ramp outdoors was 1/8 (7 degrees). The stairs consisted of two configurations: one with a height of 15 cm and the other with a height of 20 cm. The 15-cm-height stair configuration had 4 steps, with a tread depth of 25 cm and a width of 90 cm. The 20-cm-height stair configuration had 3 steps, with a tread depth of 30 cm and a width of 90 cm. The height of the walls separating the indoor and outdoor spaces was 1.1 m high to avoid blind spots for camera measurements for the markerless motion capture system. A total of 27 cameras (DSC-RX0M2, Sony Group Corporation) were deployed in the living laboratory (Figure 1).

### Participants

According to previous reliability studies [28], a minimum of 20 participants was required to ensure statistical robustness. A total of 21 healthy subjects participated in this study. Participants were recruited from staff at the National Center for Geriatrics and Gerontology. The average age of participants was 31.1 (SD 8.7) years, height was 1.67 (SD 0.09) m, and weight was 63.3 (SD 12.1) kg, with 14 males and 7 females. Those with neuromuscular or musculoskeletal disorders that could interfere with walking performance were excluded. Participants were not instructed in advance how they should dress or wear shoes in the study upon arrival. Participants provided written informed consent.

### Data Capture

Measurements were taken using 27 synchronized cameras in the living laboratory. The sampling rate of the cameras was 60 Hz. All participants performed each set of tasks on 2 days separated by an interval of at least 24 h (124.6, SD 137.5 h; maximum interval: 671.3 h, minimum interval: 24 h). Each set of tasks was performed 5 times. One set of tasks comprised (1) a 7 m level walk, (2) a ramp ascent, (3) a ramp descent, (4) a 4-step stair ascent with a height of 15 cm, and (5) a 4-step stair descent with a height of 15 cm. For all tasks, participants were instructed to start on their right foot and walk normally at their usual pace. During the measurements, participants were instructed not to use the handrails on the stairs and ramps, although the handrails were kept in place. This allowed for the assessment of their natural gait patterns when negotiating stairs and ramps, without the aid of handrails.

### Data Analysis

Video data were processed using Theia3D (version 2021.2.0.1675) to obtain 3D pose estimates of whole-body segments using default inverse kinematics and a two-degrees-of-freedom knee model (Multimedia Appendix 1). The resulting pose estimates for each body segment were exported to Visual3d (HAS-Motion) for further analysis. The gait events, initial contact (IC), and toe-off for all tasks were determined using methods described by Zeni et al [31], with quality checks performed in Visual3d. The hip-, knee-, and ankle-joint angles were calculated in the sagittal, frontal, and transverse planes and defined as the angles between the proximal and distal segments in the respective joint coordinate systems. The joint angles were time-normalized to the gait cycle, with 0% representing the IC and 100% representing the next ipsilateral IC. Representative values of interest were calculated from the gait cycle events (Table 1). However, the movements of (4) stair ascent and (5) stair descent were excluded from the analysis for the calculation of representative values because toe-off could not be determined accurately. All data were analyzed by combining the right and left sides.

**Table 1.** Gait event descriptions and abbreviations (adapted from Riazati et al [29]).

Gait event	Description
ST1	Stance phase 1, ipsilateral initial contact to mid-stance, representing the first half of the stance phase.
ST2	Stance phase 2, mid-stance to ipsilateral toe-off, representing the second half of the stance phase.
STSW	Transition from stance-to-swing phase.
RoM	The entire range of motion over the full gait cycle, presented for hip-, knee-, and ankle-joints in sagittal, frontal, and transverse planes.
MaxFlexStance	Maximum flexion angle (sagittal plane) achieved by the hip- or knee-joint during the stance phase.
MaxFlexSwing	Maximum flexion angle (sagittal plane) achieved by the hip- or knee-joint during the swing phase.
MaxFlexST1	Maximum flexion angle (sagittal plane) achieved by the knee-joint during stance phase 1.
MaxExtStance	Maximum extension angle (sagittal plane) achieved by the hip- or knee-joint during the stance phase.
MaxExtSwing	Maximum extension angle (sagittal plane) achieved by the hip- or knee-joint during swing phase.
MaxDorsiflexionST2	Maximum dorsiflexion angle (sagittal plane) achieved by the ankle-joint during the stance phase 2.
MaxPlantarflexion STSW	Maximum plantarflexion angle (sagittal plane) of the ankle-joint at stance-to-swing transition.
MaxAddDLS1	Maximum adduction angle (frontal plane) achieved by the hip-joint during double limb support phase 1.
MaxAbdSwing	Maximum abduction angle (frontal plane) achieved by the hip-joint during the swing phase.
MaxVarusSwing	Maximum varus angle (frontal plane) achieved by the knee-joint in the swing phase.
MaxInvSwing	Maximum inversion angle (frontal plane) achieved by the ankle-joint during the swing phase.
MaxEvST1	Maximum eversion angle (frontal plane) achieved by the ankle-joint during the stance phase 1.
MaxIntRotST1	Maximum internal rotation (transverse plane) achieved by the hip- or ankle-joint during the stance phase 1.
MaxExtRotST2	Maximum external rotation (transverse plane) achieved by the hip- or ankle-joint during the stance phase 2.
MaxExtRotSwing	Maximum external rotation (transverse plane) achieved by the hip- or ankle-joint during the swing phase.
AnkleInitialContact	Ankle-joint angle (sagittal or frontal plane) at initial contact.

### Statistical Analysis

Statistical analysis was performed using the same approach as Riazati et al [29]. First, error evaluation was performed using the approach described in Kanko et al [18], where root-mean-square differences (RMSDs) were calculated to assess intersession differences of all curves for the 3 joints and plane of motion. RMSD analysis quantifies the magnitude of differences between sessions, with lower values indicating greater reproducibility between measurements. For each session, the average RMSD was calculated using the ensemble averages of the participants’ within-session data. Second, a reliability evaluation was performed using the standard error of measurement (SEM) of representative values within gait cycles [32]. SEM provides the magnitude of measurement error in degrees between different days, allowing comparison with clinical acceptability criteria [28]. SEM was calculated using the following equation.


(1) $$ICC(3,10)=\frac{MSB-MSW}{MSB+\frac{(k-1)MSW}{k}}$$

(2) $$SEM=SD\times \sqrt{1-ICC}$$

Here MSB indicates the between-subjects mean square, MSW indicates the within-subjects mean square, and k is the number of trials. SD indicates standard deviation of the data of all subjects. The SEM provides an error value in the same units as the measurement. Measurement errors between 2° and 5° are considered acceptable [28]. All statistical analysis was performed in MATLAB (version 2017a; MathWorks) and Python (version 3.8; Python Software Foundation).

### Ethical Considerations

The study was approved by the Committee on Ethics and Conflict of Interest of the National Center for Geriatrics and Gerontology (acceptance 1636).

## Results

### RMSD, SEM, and RoM Values

**Figure 2.**
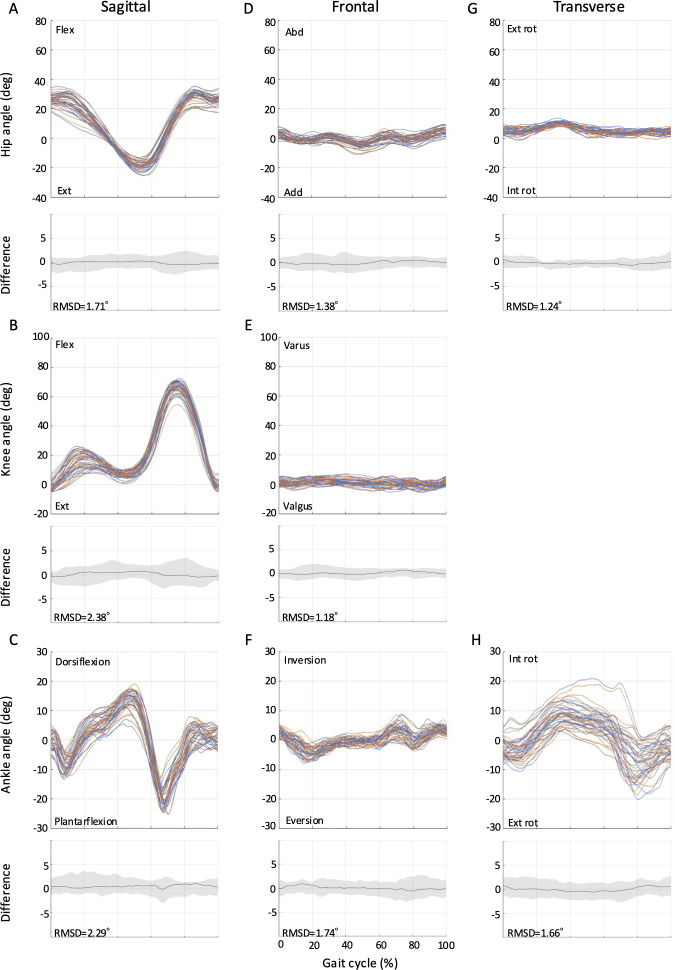
Between-session reproducibility of full-curve analysis of subject-average joint angles during level walking. Data description joint angles for hip (row 1), knee (row 2), and ankle (row 3) in the sagittal (A-C), frontal (D-F), and transverse planes (G-H). Each curve represents the ensemble average of each individual’s trials on Day 1 (blue lines) and Day 2 (orange lines). The average RMSD across all subjects is shown below the respective joint angle plot; across all joints and planes of motion, the largest root mean square difference (RMSD) was 2.38°. deg: degree; Ext: extension; Abd: abduction; Add: adduction; Ext rot: external rotation; Int rot: internal rotation; RMSD: root mean square difference.

**Figure 3.**
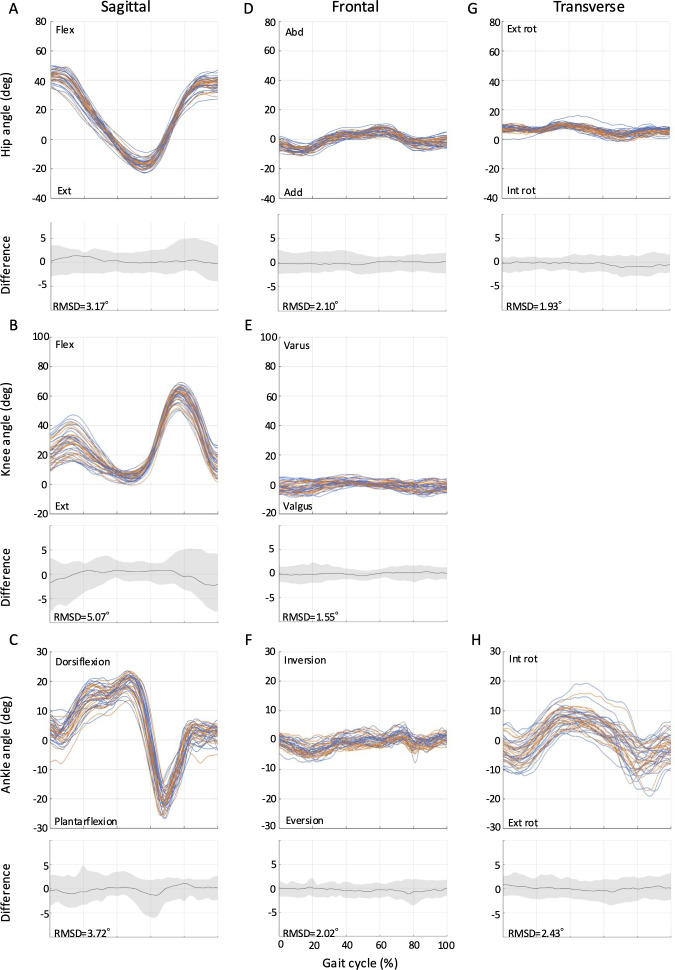
Between-session reproducibility of full-curve analysis of subject-average joint angles during ramp ascent. Data description joint angles for hip (row 1), knee (row 2), and ankle (row 3) in the sagittal (A-C), frontal (D-F), and transverse planes (G-H). Each curve represents the ensemble average of each individual’s trials on Day 1 (blue lines) and Day 2 (orange lines). The average RMSD across all subjects is shown below the respective joint angle plot; across all joints and planes of motion, the largest RMSD was 5.07°. deg: degree; Ext: extension; Abd: abduction; Add: adduction; Ext rot: external rotation; Int rot: internal rotation; RMSD: root mean square difference.

**Figure 4.**
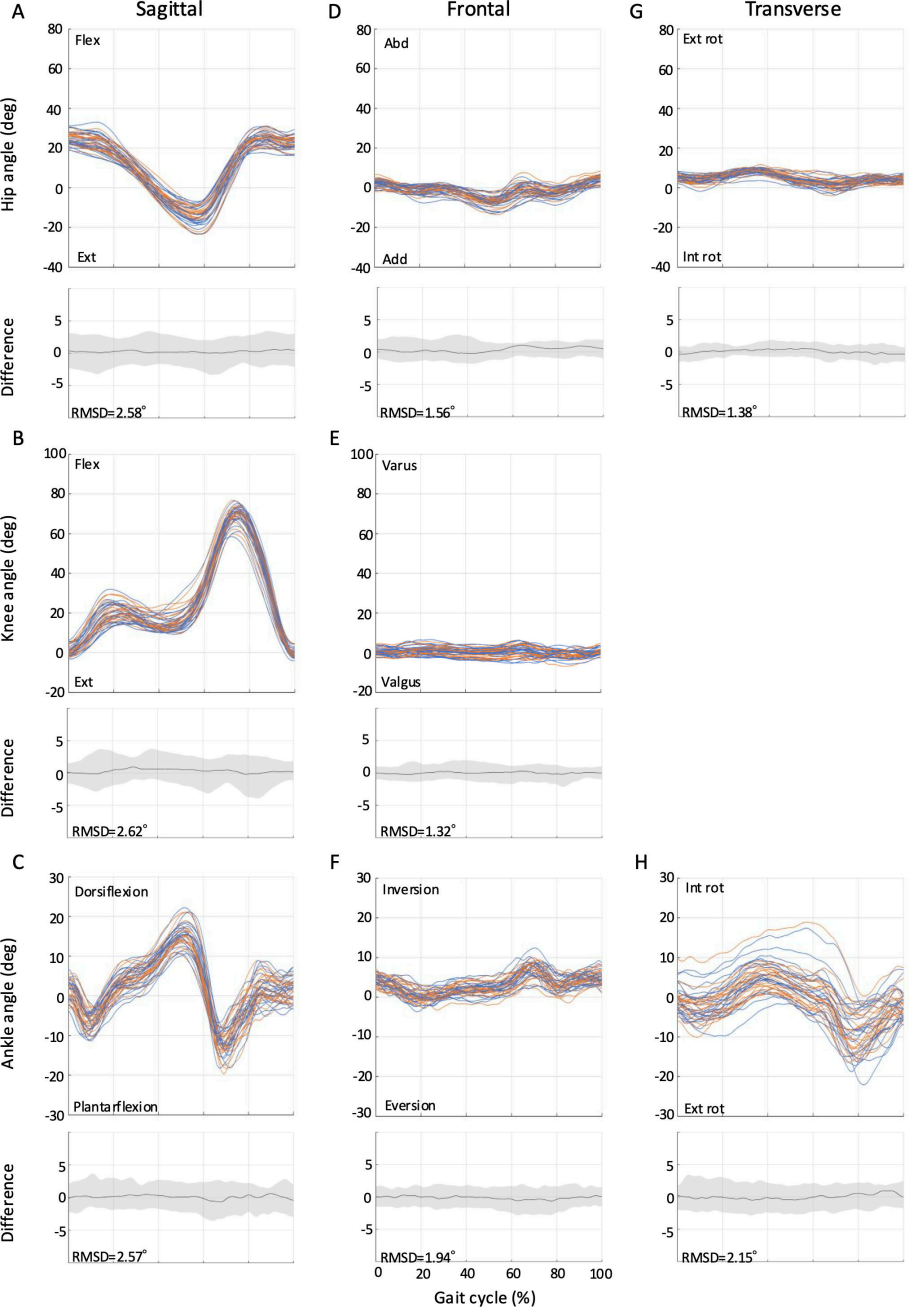
Between-session reproducibility of full-curve analysis of subject-average joint angles during ramp descent. Data description joint angles for hip (row 1), knee (row 2), and ankle (row 3) in the sagittal (A-C), frontal (D-F), and transverse planes (G-H). Each curve represents the ensemble average of each individual’s trials on Day 1 (blue lines) and Day 2 (orange lines). The average RMSD across all subjects is shown below the respective joint angle plot; across all joints and planes of motion, the largest RMSD was 2.62°. deg: degree; Ext: extension; Abd: abduction; Add: adduction; Ext rot: external rotation; Int rot: internal rotation; RMSD: root mean square difference.

**Figure 5.**
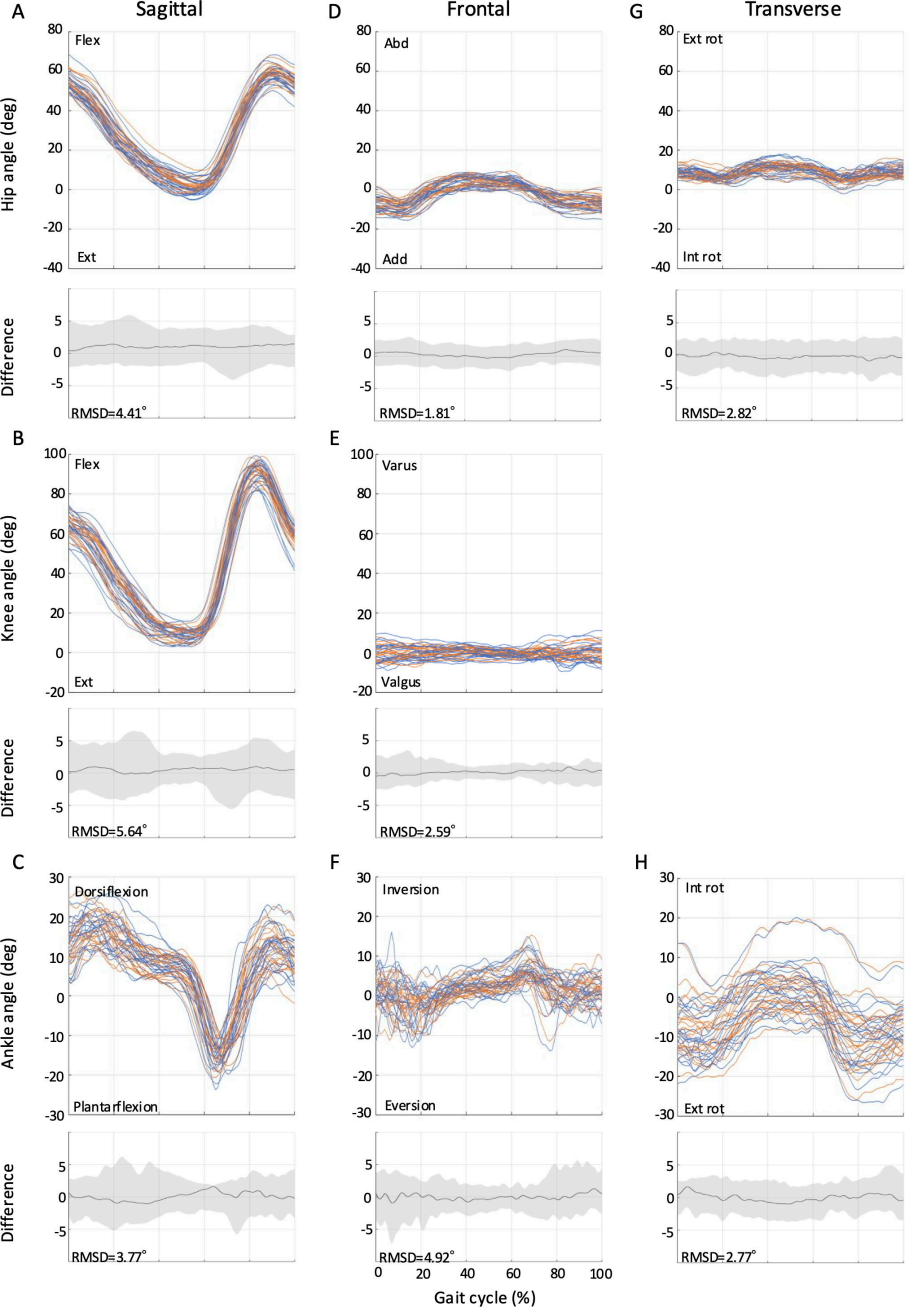
Between-session reproducibility of full-curve analysis of subject-average joint angles during stair ascent. Data description joint angles for hip (row 1), knee (row 2), and ankle (row 3) in the sagittal (A-C), frontal (D-F), and transverse planes (G-H). Each curve represents the ensemble average of each individual’s trials on Day 1 (blue lines) and Day 2 (orange lines). The average RMSD across all subjects is shown below the respective joint angle plot; across all joints and planes of motion, the largest RMSD was 5.64°. deg: degree; Ext: extension; Abd: abduction; Add: adduction; Ext rot: external rotation; Int rot: internal rotation; RMSD: root mean square difference.

**Figure 6.**
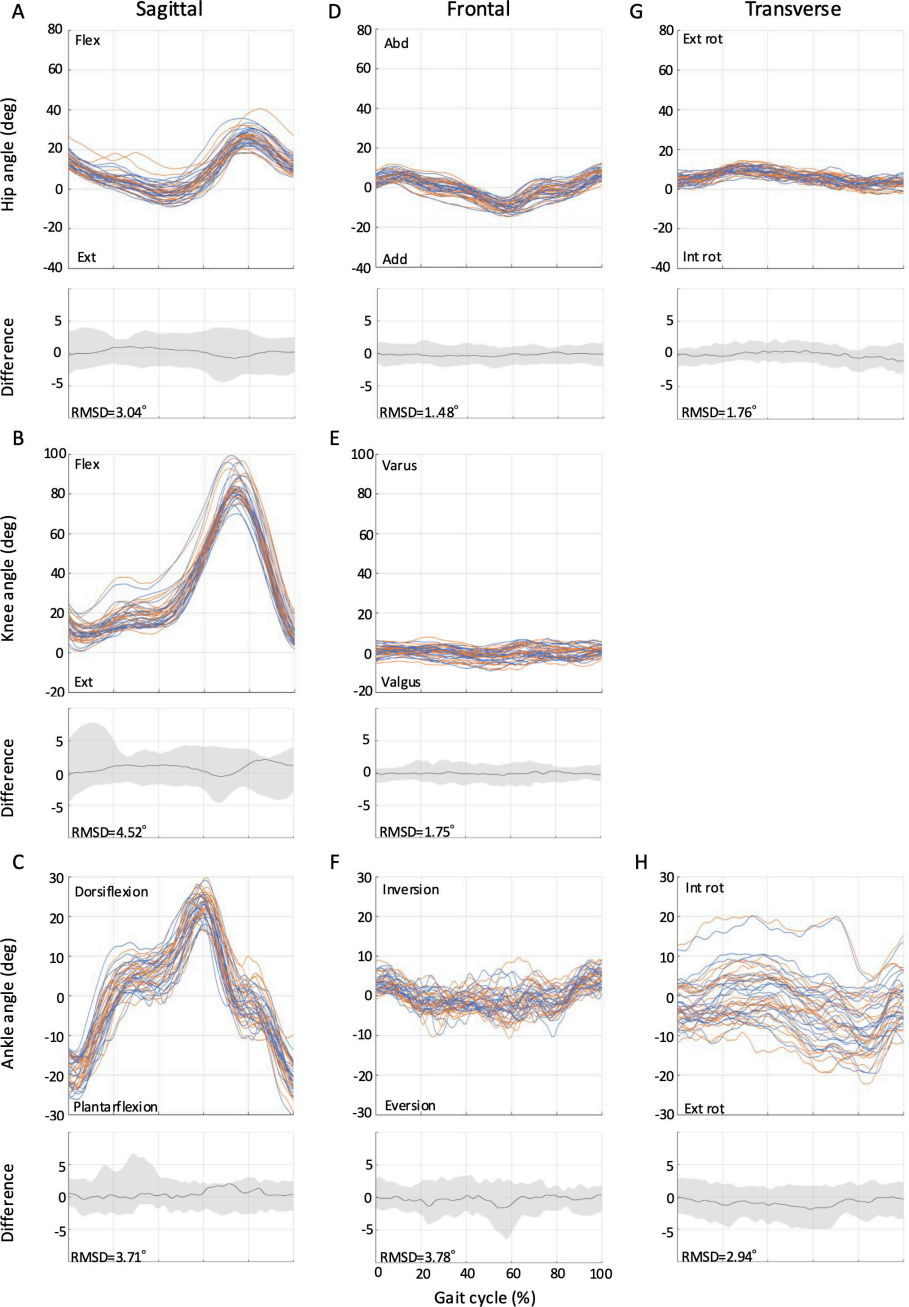
Between-session reproducibility of full-curve analysis of subject-average joint angles during stair descent. Data description joint angles for hip (row 1), knee (row 2), and ankle (row 3) in the sagittal (A-C), frontal (D-F), and transverse planes (G-H). Each curve represents the ensemble average of each individual’s trials on Day 1 (blue lines) and Day 2 (orange lines). The average RMSD across all subjects is shown below the respective joint angle plot; across all joints and planes of motion, the largest RMSD was 4.52°. deg: degree; Ext: extension; Abd: abduction; Add: adduction; Ext rot: external rotation; Int rot: internal rotation; RMSD: root mean square difference.

The RMSDs from full-curve analysis of the kinematics for all tasks (ie, level walking, ramp ascent, ramp descent, stair ascent, and stair descent) are shown in Figures 2-6. The SEMs of representative values within gait cycles are presented in Tables 2-4. Measurement errors as given by SEM and RMSD were <5° for the majority of variables, and were thus deemed acceptable [28]. Representative range of motion (RoM) for the hip-, knee-, and ankle-joint was also calculated and comparable values were extracted for most tasks over the study period. A summary of the RMSD, SEM, and RoM values for each task is given below.

**Table 2.** Level walking, mean (SD) for Day 1 and Day 2, and standard error of measurement (SEM).

Planes and joints parameters	Day 1, mean (SD)	Day 2, mean (SD)	SEM^a^
Sagittal			
Hip (degrees)			
RoM^b^	48.9 (5.7)	49.6 (5.8)	1.56
MaXFlexstance	27.0 (4.2)	27.3 (4.5)	1.30
MaxFlexswing	29.4 (4.0)	29.9 (4.1)	3.23
MaxExtstance	18.8 (3.5)	19.1 (3.9)	1.19
Knee (degrees)			
RoM	68.8 (4.6)	68.9 (4.8)	1.25
MaxFlexst1	18.5 (5.3)	17.7 (6.0)	1.70
MaxFlexswing	66.7 (4.7)	66.7 (5.2)	1.12
MaxExtstance	1.4 (3.3)	1.4 (3.2)	0.82
Ankle (degrees)			
RoM	36.6 (4.3)	36.3 (4.8)	1.60
InitialContact	0.3 (4.3)	–0.3 (4.3)	1.35
MaxDorsiflexionst2	14.9 (3.5)	14.3 (4.2)	1.13
MaxPlanterflexionststsw	21.5 (4.0)	21.9 (4.7)	1.42
Frontal			
Hip (degrees)			
RoM	12.4 (3.5)	12.3 (3.2)	1.08
MaxAddDLS1	1.8 (3.4)	2.1 (3.9)	0.89
MaxAbdswing	4.9 (3.9)	4.1.(4.3)	0.86
Knee (degrees)			
RoM	8.1 (2.2)	8.6 (2.4)	1.00
MaxVarusswing	3.9 (2.8)	3.6 (3.0)	0.71
Ankle (degrees)			
RoM	13.5 (4.0)	14.4 (3.6)	1.89
InitialContact	3.1 (4.6)	3.0 (4.7)	1.47
MaxInvswing	7.7 (5.7)	8.0 (5.3)	1.39
MaxEvst1	5.6 (4.7)	6.3 (4.8)	1.25
Transverse			
Hip (degrees)			
RoM	11.4 (2.7)	12.3 (3.0)	1.01
MaxIntRotst1	2.6 (2.9)	1.7 (3.5)	1.03
MaxRotst2	11.1 (2.6)	10.6 (3.1)	1.00
Ankle (degrees)			
RoM	21.1 (4.3)	21.8 (4.7)	1.47
MaxIntRotst1	8.4 (4.7)	8.9 (4.9)	1.27
MaxExtRotswing	4.1 (6.3)	4.6 (4.2)	1.51

a SEM: standard error of measurement.

b RoM: range of motion.

**Table 3.** Ramp ascent, mean (SD) for Day 1 and Day 2, and standard error of measurement (SEM).

Planes and joints parameters	Day 1, mean (SD)	Day 2, mean (SD)	SEM^a^
Sagittal			
Hip (degrees)			
RoM^b^	62.7 (7.7)	61.3 (9.0)	4.20
MaXFlexstance	44.7 (5.4)	43.4 (6.7)	3.01
MaxFlexswing	39.5 (7.1)	40.6 (7.3)	2.73
MaxExtstance	17.8 (3.8)	17.5 (4.6)	2.23
Knee (degrees)			
RoM	59.4 (5.3)	58.8 (6.7)	2.61
MaxFlexst1	30.8 (9.8)	29.8 (10.6)	4.37
MaxFlexswing	63.7 (5.7)	63.5 (6.8)	2.85
MaxExtstance	4.2 (3.1)	3.7 (3.0)	1.40
Ankle (degrees)			
RoM	44.7 (5.2)	43.7 (6.0)	2.63
InitialContact	2.5 (5.0)	2.9 (4.5)	2.31
MaxDorsiflexionst2	21.0 (3.6)	20.5 (3.7)	1.98
MaxPlanterflexionststsw	23.4 (4.5)	22.9 (5.2)	2.18
Frontal			
Hip (degrees)			
RoM	16.1 (4.0)	15.8 (4.0)	2.07
MaxAddDLS1	8.3 (4.7)	7.1 (5.2)	1.48
MaxAbdswing	5.2 (4.8)	4.5 (4.4)	1.28
Knee (degrees)			
RoM	9.3 (3.2)	9.7 (3.6)	1.47
MaxVarusswing	2.7 (3.6)	2.4 (3.8)	1.50
Ankle (degrees)			
RoM	12.2 (4.8)	12.4 (4.4)	2.23
InitialContact	0.8 (5.4)	0.7 (5.2)	1.45
MaxInvswing	6.1 (6.6)	6.4 (5.9)	1.94
MaxEvst1	5.6 (5.9)	5.8 (5.9)	1.83
Transverse			
Hip (degrees)			
RoM	11.8 (2.9)	15.8 (2.7)	1.57
MaxIntRotst1	3.9 (2.7)	4.2 (2.8)	1.56
MaxRotst2	10.9 (2.6)	11.0 (3.3)	1.56
Ankle (degrees)			
RoM	19.6 (4.1)	20.0 (4.5)	2.10
MaxIntRotst1	7.7 (5.6)	7.3 (5.9)	2.50
MaxExtRotswing	2.4 (6.0)	2.5 (6.5)	2.57

a SEM: standard error of measurement.

b RoM: range of motion.

**Table 4.** Ramp descent, mean (SD) for Day 1 and Day 2, and standard error of measurement (SEM).

Planes and joints parameters	Day 1, mean (SD)	Day 2, mean (SD)	SEM^a^
Sagittal			
Hip (degrees)			
RoM^b^	42.2 (6.5)	42.0 (7.0)	2.29
MaXFlexstance	25.5 (5.1)	24.9 (4.8)	1.86
MaxFlexswing	26.6 (4.1)	26.0 (4.0)	1.74
MaxExtstance	14.6 (5.2)	15.1 (5.6)	1.85
Knee (degrees)			
RoM	72.0 (4.9)	72.0 (5.4)	2.15
MaxFlexst1	22.6 (6.6)	21.5 (6.4)	2.12
MaxFlexswing	70.2 (5.2)	70.3 (6.3)	1.57
MaxExtstance	0.4 (3.6)	0.2 (3.4)	1.12
Ankle (degrees)			
RoM	33.4 (5.9)	32.7 (5.5)	2.42
InitialContact	1.3 (3.9)	1.7 (4.3)	1.53
MaxDorsiflexionst2	17.0 (3.9)	16.9 (4.1)	1.67
MaxPlanterflexionststsw	16.4 (5.0)	15.6 (4.3)	2.16
Frontal			
Hip (degrees)			
RoM	14.1 (4.0)	13.6 (3.5)	1.62
MaxAddDLS1	–1.0 (5.2)	0.4 (5.5)	1.09
MaxAbdswing	5.9 (4.6)	5.4 (4.5)	0.97
Knee (degrees)			
RoM	8.1 (2.4)	7.8 (2.1)	1.49
MaxVarusswing	3.2 (2.9)	2.9 (2.9)	1.03
Ankle (degrees)			
RoM	12.9 (4.0)	12.5 (3.7)	1.55
InitialContact	4.4 (4.9)	4.3 (4.8)	1.47
MaxInvswing	7.9 (7.0)	8.2 (6.8)	1.25
MaxEvst1	2.3 (5.1)	2.0 (5.2)	1.50
Transverse			
Hip (degrees)			
RoM	10.8 (2.7)	13.6 (2.7)	1.17
MaxIntRotst1	2.5 (3.1)	2.7 (3.1)	1.62
MaxRotst2	9.7 (3.0)	9.4 (3.0)	1.42
Ankle (degrees)			
RoM	19.1 (4.3)	19.7 (4.9)	1.83
MaxIntRotst1	5.1 (6.1)	5.3 (5.6)	1.67
MaxExtRotswing	3.7 (6.7)	3.1 (6.0)	1.51

a SEM: standard error of measurement.

b RoM: range of motion.

### Level Walking

The RMSD values ranged from 1.2° to 2.4° for level walking (Figure 2). For the same task, the SEMs ranged from 0.82° to 3.23° in the sagittal plane, 0.71° to 1.89° in the frontal plane, and 1.01° to 1.51° in the transverse plane (Table 2). Sagittal plane RoM values of 48.9° (SD 5.7°; Day 1) and 49.6° (SD 5.8°; Day 2) for the hip-joint, 68.8° (4.6°; Day 1) and 68.9° (SD 4.8°; Day 2) for the knee-joint, and 36.6° (SD 4.3°; Day 1) and 36.3° (SD 4.8°; Day 2) for the ankle-joint were obtained.

### Ramp Ascent

The RMSD ranged from 1.6° to 5.1° for ramp ascent (Figure 3). The highest RMSD was observed for knee-joint flexion-extension angle as 5.1°. Otherwise, the RMSD values were less than 5°. The SEM ranged from 1.40° to 4.37° in the sagittal plane, 1.45° to 2.07° in the frontal plane, and 1.57° to 2.57° in the transverse plane for ramp ascent (Table 3). During ramp ascent, joint angle changes were larger compared to level walking. The sagittal plane RoM of the hip-joint was 62.7° (SD 7.7°; Day 1) and 61.3° (SD 9.0°; Day 2), which was 13.8° (Day 1) and 11.7° (Day 2) greater than level walking. The knee-joint flexion angle during early stance was 30.8° (SD 9.8°; Day 1) and 29.8° (SD 10.6°; Day 2), an increase of 12.3° (Day 1) and 12.1° (Day 2). In addition, the ankle-joint dorsiflexion angle during stance was 21.0° (SD 3.6°; Day 1) and 20.5° (SD 3.7°; Day 2), an increase of 6.1° (Day 1) and 6.2° (Day 2).

### Ramp Descent

The RMSDs ranged from 1.3° to 2.6° for ramp descent (Figure 4), while the SEM values ranged from 1.12° to 2.42° in the sagittal plane, 0.97° to 1.62° in the frontal plane, and 1.17° to 1.83° in the transverse plane (Table 4). During ramp descent, joint angle changes were also compared with level walking. The hip-joint RoM was 42.2° (SD 6.5°; Day 1) and 42.0° (SD 7.0°; Day 2), a decrease of 6.7° (Day 1) and 7.6° (Day 2) from level walking. However, the knee-joint RoM increased by 3.2° (Day 1) and 3.1° (Day 2) to 72.0° (SD 4.9°; Day 1) and 72.0° (SD 5.4°; Day 2). The maximum ankle-joint plantar flexion angle decreased by 5.1° (Day 1) and 6.3° (Day 2) to 16.4° (SD 5.0°; Day 1) and 15.6° (SD 4.3°; Day 2).

### Stair Ascent

RMSDs ranged from 1.8° to 5.7° for stair ascent (Figure 5). The RMSD of the knee-joint flexion-extension angle showed the highest value at 5.7°, but otherwise the RMSD values were less than 5°. The hip-joint RoM was 59.5° (SD 5.1°; Day 1) and 60.2 (SD 6.1°; Day 2), an increase of 10.6° (Day 1) and 10.6° (Day 2) compared to level walking. The knee-joint RoM was 86.4° (SD 6.2°; Day 1) and 86.1° (SD 7.4°; Day 2), an increase of 17.6° (Day 1) and 17.2° (Day 2) over level walking. The ankle-joint RoM was 40.0° (SD 8.3°; Day 1) and 41.6° (SD 7.8°; Day 2), which was 3.4° (Day 1) and 5.3° (Day 2) greater than level walking.

### Stair Descent

The RMSD values ranged from 1.5° to 4.5° for stair descent (Figure 6). The hip-joint RoM was 30.1° (SD 3.6°; Day 1) and 31.0° (SD 4.4°; Day 2), a decrease of 18.8° (Day 1) and 18.6° (Day 2) compared to level walking. The knee-joint RoM was 81.9° (SD 7.3°; Day 1) and 81.5° (SD 7.8°; Day 2), which was 13.1° (Day 1) and 12.6° (Day 2) greater than for level walking. The ankle-joint RoM was 50.9° (SD 6.6°; Day 1) and 51.1° (SD 6.0°; Day 2), an increase of 14.3° (Day 1) and 14.8° (Day 2) over level walking

## Discussion

### Principal Results and Comprehensive Analysis

This study showed that the Theia3D markerless motion capture provides reliable joint angle measurements across a wide range of locomotion tasks, including level walking, ramp ascent, ramp descent, stair ascent, and stair descent in the living laboratory environment. This is evidenced by the low SEM and RMSD values extracted by full-curve analysis of the kinematics and their intersession differences. The SEM values for hip-, knee-, and ankle-joint angles were less than 5°, and the RMSD values were also generally below 5°. According to McGinley et al [28], measurement errors less than 5° are considered acceptable for clinical interpretation, indicating good agreement between the joint angle waveforms across days in our study. Previous research has examined the dependability of the Theia3D system in relation to level walking; however, to the best of our knowledge, this is the first study to assess its absolute reliability for stair and ramp walking tasks, and therefore, there is nothing in the literature with which we can compare these results. Our findings for level walking, however, are consistent with those of previous studies that investigated the reliability of the Theia3D system. For example, our SEM values for level walking (0.82° to 3.23°) are comparable to those reported by Riazati et al [29] (0.91° to 4.95°). Similarly, our RMSD values for level walking (1.2° to 2.4°) are also consistent with those of the same authors [29] (0.96° to 3.56°). Therefore, the markerless motion capture system in our living laboratory setting provides the same level of reliability as previous studies, suggesting that it can be applied not only to level walking but also to various other walking situations.

Notably, this study could extend the application of the markerless motion capture by demonstrating its absolute reliability across a broader range of locomotion tasks commonly encountered in daily life, including ramps and stairs. The SEM (1.40° to 4.37° for ascent; 0.97° to 2.42° for descent) and RMSD values (1.6° to 5.1° for ascent; 1.3° to 2.6° for descent) for these tasks suggest that the Theia3D system can reliably measure joint angles during ramp ascent and descent. Furthermore, this study was the first to report RMSD values for stair gait using the markerless motion capture system. The RMSD values for stair ascent (1.8° to 5.7°) and descent (1.4° to 4.5°) were slightly higher than those for level walking and ramp gait, which may be attributed to the greater complexity and variability of stair gait tasks [21]. Nonetheless, the majority of RMSD values for stair gait remained within acceptable limits, demonstrating the potential of the Theia3D system for analyzing these complex gait patterns in the living laboratory setting. Furthermore, the joint angles observed during stair ascent and descent showed patterns similar to those reported in previous marker-based motion capture studies [33,34], further supporting the validity of our markerless measurements in capturing movement adaptations during complex tasks.

### Comparison With Previous Work

As reported in previous studies [35], the versatility of the Theia3D system in performing reliable measurements across a range of environments is particularly important considering the challenges traditionally faced by motion capture systems. One of these challenges is the difficulty encountered when accuracy is reduced due to imperfections in the experimental set-up, such as camera placement and the presence of obstacles within the measurement area [18,19,36]. The living laboratory has handrails on both sides of the stairs and ramps, as well as a 110-cm wall and pillars separating the indoor and outdoor spaces, which are considered poor measurement environments. Despite these challenges, the SEM and RMSD values in this study were low. This suggests that the Theia3D system can maintain high reproducibility and reliably measure joint angles, probably by complementing any blind spots of the 27 cameras, even in the presence of environmental barriers. Furthermore, Figure 1 shows that the suspension of cameras from the ceiling allowed measurements to be made without interfering with the range of human movement. The possibility of more efficient placement of environmental barriers and cameras need to be further explored, but our markerless motion capture system in the living laboratory opens up new possibilities for gait analysis in real-world settings and will provide valuable data to researchers and clinicians.

### Implications and Applications of the Result

The uniqueness and novelty of this study lies in the fact that various gait data, such as ramp and stair ascent and descent, could be reliably obtained under conditions similar to those in everyday life. A system capable of precisely measuring the differences between such walking activities is crucial for understanding how an individual’s gait pattern adapts to the diverse terrains encountered in daily life. For example, while joint angle variations are relatively small during level walking [37], joint angles may change significantly during ramp and stair walking in response to the demands of the task. Previous studies have revealed various adaptation and walking strategies that individuals use to cope with uneven terrain and stairs [38,39]. Further accumulation of longitudinal data on gait adaptation using the living laboratory fitted with various environmental barriers such as uneven terrain and stairs may provide new evidence for some aspects of the adaptive strategy mechanism.

Our results showed that, compared to level walking, ramp ascent increased the range of hip-joint motion by 13.8°, knee-joint flexion angle during early stance by 12.3°, and ankle-joint dorsiflexion angle during stance by 6.1°. In contrast, ramp descent decreased the range of hip-joint motion by 6.7° and maximum ankle-joint plantar flexion angle by 5.1°, while increasing the range of knee-joint motion by 3.2°. These findings are consistent with the increased hip- and knee-joint flexion angles during ramp walking reported by Lay et al [40] and the increased knee-joint flexion and decreased ankle-joint plantar flexion angles during ramp descent observed by McIntosh et al [41]. These joint angle adaptations are thought to reflect strategies for controlling the vertical movement of the body’s center of mass during ramp ascent and descent. Furthermore, joint angle adaptations were even more pronounced during stair climbing compared to level walking. During stair ascent, the range of hip-joint motion increased by 10.6°, the range of knee-joint motion by 17.6°, and the range of ankle-joint motion by 3.4° compared to level walking. During stair descent, the range of hip-joint motion decreased by 18.8°, while the range of knee-joint motion increased by 13.1° and the range of ankle-joint motion by 14.3° compared to level walking. These results are consistent with the increased hip- and knee-joint flexion angles during stair ascent reported by Riener et al [33] and the decreased hip-joint flexion, increased knee-joint flexion and increased ankle-joint plantar flexion angles during stair descent observed by Protopapadaki et al [42]. Interestingly, this showed that RMSD values for stair ascent and descent were slightly higher at 1.8‐5.7° and 1.5‐4.5°, respectively, compared to level walking (1.2‐2.4°), which may reflect the greater demands on balance and stability of this task [43].

In the future, it should be possible to apply the markerless motion capture system in the living laboratory to a wide range of research fields. For example, it could be used to investigate how environmental factors such as stair height and inadequate handrails affect changes in movement patterns between individuals [44]. An application might be where an individual, who is unable to perform certain essential ADLs in the home environment, might be able to simulate these tasks in the living laboratory before being discharged from the hospital [45]. It may thus be possible to assess whether this difficulty is due to a person’s ability to adapt to the home environment. Such an approach could be useful in designing real-life living environments and improving welfare services to support independent living for older adults and people with disabilities. Furthermore, the Theia3D system is likely not only useful for evaluating the effectiveness of rehabilitation and training for patients with gait disorders but might also be applied in the fields of robotics and bioengineering to develop robots with adaptive walking mechanisms and to design walking support devices [46,47]. Our previous study demonstrated that this system can provide data for optimal selection of assistive robots by measuring changes in center of mass during robot use [20]. In this study, reproducibility was verified for the walking movements studied, as walking offers a relatively consistent movement pattern for reliability testing. However, expanding the scope of the analysis beyond gait to encompass more intricate ADLs, such as standing, sitting, and lifting objects, represents a key avenue for future research [48]. This could contribute to the growing body of ADL movement databases and enable various applications that consider differences in architectural and lifestyle patterns across different cultures. Furthermore, the importance of robot technology in supporting not only walking movements but also transferring and releasing a person from a bed has been described in the nursing and medical fields [49-51]. The living laboratory could provide information to further develop such assistive robots in the future.

### Limitations

The study has several limitations. First, although previous studies have shown that markerless motion capture systems are less susceptible to the influence of subjects’ clothing [52,53], this study did not directly verify this point. We did not standardize the subjects’ clothing, but 13 out of 21 subjects wore the same clothing on both days. This might have contributed to the good results obtained in this study. Future studies should conduct measurements under various clothing conditions to investigate the impact of clothing in more detail. The second limitation is that the algorithm used to detect foot contact and foot lift events may not be applicable to all types of movements. The current algorithm relies on the positional relationship of the feet to determine foot contact and lift, which could make it challenging to use with patients who have conditions that cause irregular gait patterns. The third limitation is that the subjects in this study were all healthy young adults (14 males and 7 females), and no verification was conducted on older adults or patients with gait disorders. The gender distribution and ethnic homogeneity of our participants limit the generalizability of our findings to other populations. The advantages of markerless systems are expected to be greater for older adults and patients with gait disorders; therefore, future studies should include a variety of subjects to verify whether the results can be generalized across different age groups, genders, and ethnicities. The other limitation is the absence of concurrent validation with a marker-based motion capture system, which is considered the gold standard. Previous studies have demonstrated the importance of simultaneous comparison between two motion capture systems to evaluate systematic and random errors [54]. While such comparison would have provided additional validation through systematic error analysis, our study focused on the between-day reliability of measurements in a living laboratory setting. At last, while we used both RMSD and SEM to assess reliability, clinical application requires consideration of the Minimal Clinically Important Difference (MCID). Establishing MCID thresholds in future studies will be essential for determining whether observed changes in gait parameters are meaningful in clinical practice.

### Conclusions

This study used the Theia3D markerless motion capture system in a living laboratory environment to evaluate the absolute reliability of joint angles during the various gait tasks, including level walking, ramp and stair ascent and descent using representative RMSD and SEM values. The results showed that our system could reliably measure joint angles during these tasks in a living laboratory setting. The observed joint angle adaptations provide valuable insight into the strategies individuals use to maintain stability and prevent falls when walking. These findings highlight the potential of markerless motion capture technology in real-world gait assessment. This evaluation system in living laboratories can be used more widely to explore rehabilitation strategies and robotics applications in a variety of subject groups, including older adults and patients with gait impairments.

## Supplementary material

10.2196/66886 Multimedia Appendix 1 Markerless processing.

## Abbreviations

ADLs: activities of daily living
IC: initial contact
MCID: minimal clinically important difference
RMSD: root mean square difference
RoM: range of motion
SEM: standard error of measurement

## References

[1] White DK, Neogi T, Nevitt MC (2013). Trajectories of gait speed predict mortality in well-functioning older adults: the Health, Aging and Body Composition study. J Gerontol A Biol Sci Med Sci.

[2] Roush J, Heick J, Hawk T, Eurek D, Wallis A, Kiflu D (2021). Agreement in walking speed measured using four different outcome measures: 6-meter walk test, 10-meter walk test, 2-minute walk test, and 6-minute walk test. IJAHSP.

[3] Montero-Odasso M, Speechley M, Muir-Hunter SW (2020). Dual decline in gait speed and cognition is associated with future dementia: evidence for a phenotype. Age Ageing.

[4] Stanaway FF, Gnjidic D, Blyth FM (2011). How fast does the Grim Reaper walk? Receiver operating characteristics curve analysis in healthy men aged 70 and over. BMJ.

[5] Khanittanuphong P, Tipchatyotin S (2017). Correlation of the gait speed with the quality of life and the quality of life classified according to speed-based community ambulation in Thai stroke survivors. NeuroRehabilitation.

[6] Lord S, Galna B, Verghese J, Coleman S, Burn D, Rochester L (2013). Independent domains of gait in older adults and associated motor and nonmotor attributes: validation of a factor analysis approach. J Gerontol A Biol Sci Med Sci.

[7] Anang N, Jailani R, Tahir NM, Manaf H, Mustafah N Analysis of kinematic gait parameters in chronic stroke survivors.

[8] Schmitt D, Vap A, Queen RM (2015). Effect of end-stage hip, knee, and ankle osteoarthritis on walking mechanics. Gait Posture.

[9] Dollar AM, Herr H (2008). Lower extremity exoskeletons and active orthoses: challenges and state-of-the-art. IEEE Trans Robot.

[10] Hussain S, Xie SQ, Liu G (2011). Robot assisted treadmill training: mechanisms and training strategies. Med Eng Phys.

[11] Cockcroft J, Louw Q, Baker R (2016). Proximal placement of lateral thigh skin markers reduces soft tissue artefact during normal gait using the Conventional Gait Model. Comput Methods Biomech Biomed Engin.

[12] Cronin NJ (2021). Using deep neural networks for kinematic analysis: challenges and opportunities. J Biomech.

[13] Yamamoto M, Shimatani K, Hasegawa M, Kurita Y, Ishige Y, Takemura H (2021). Accuracy of temporo-spatial and lower limb joint kinematics parameters using openpose for various gait patterns with orthosis. IEEE Trans Neural Syst Rehabil Eng.

[14] Needham L, Evans M, Wade L (2022). The development and evaluation of a fully automated markerless motion capture workflow. J Biomech.

[15] Abbondanza P, Giancola S, Sala R, Tarabini M (2017). Wireless Mobile Communication and Healthcare.

[16] Cao Z, Hidalgo G, Simon T, Wei SE, Sheikh Y (2021). OpenPose: realtime multi-person 2D pose estimation using part affinity fields. IEEE Trans Pattern Anal Mach Intell.

[17] Van Hooren B, Pecasse N, Meijer K, Essers JMN (2023). The accuracy of markerless motion capture combined with computer vision techniques for measuring running kinematics. Scand J Med Sci Sports.

[18] Kanko RM, Laende EK, Davis EM, Selbie WS, Deluzio KJ (2021). Concurrent assessment of gait kinematics using marker-based and markerless motion capture. J Biomech.

[19] Kanko RM, Laende EK, Strutzenberger G (2021). Assessment of spatiotemporal gait parameters using a deep learning algorithm-based markerless motion capture system. J Biomech.

[20] Kato K, Yoshimi T, Shimotori D (2024). Development of a living laboratory to verify assistive technology in simulated indoor and outdoor spaces. JACIII.

[21] Startzell JK, Owens DA, Mulfinger LM, Cavanagh PR (2000). Stair negotiation in older people: a review. J Am Geriatr Soc.

[22] Gottschall JS, Okorokov DY, Okita N, Stern KA (2011). Walking strategies during the transition between level and hill surfaces. J Appl Biomech.

[23] Sheehan RC, Gottschall JS (2012). At similar angles, slope walking has a greater fall risk than stair walking. Appl Ergon.

[24] Horak FB (2006). Postural orientation and equilibrium: what do we need to know about neural control of balance to prevent falls?. Age Ageing.

[25] Reeves ND, Spanjaard M, Mohagheghi AA, Baltzopoulos V, Maganaris CN (2008). The demands of stair descent relative to maximum capacities in elderly and young adults. J Electromyogr Kinesiol.

[26] Reeves ND, Spanjaard M, Mohagheghi AA, Baltzopoulos V, Maganaris CN (2009). Older adults employ alternative strategies to operate within their maximum capabilities when ascending stairs. J Electromyogr Kinesiol.

[27] Mahoney FI, Barthel DW (1965). Functional evaluation: The Barthel Index: A simple index of independence useful in scoring improvement in the rehabilitation of the chronically ill. Md State Med J.

[28] McGinley JL, Baker R, Wolfe R, Morris ME (2009). The reliability of three-dimensional kinematic gait measurements: a systematic review. Gait Posture.

[29] Riazati S, McGuirk TE, Perry ES, Sihanath WB, Patten C (2022). Absolute reliability of gait parameters acquired with markerless motion capture in living domains. Front Hum Neurosci.

[30] Ministry of Land IT, Tourism (2024). Ordinance for enforcement of the Building Standards Act. Government of Japan.

[31] Zeni JA, Richards JG, Higginson JS (2008). Two simple methods for determining gait events during treadmill and overground walking using kinematic data. Gait Posture.

[32] Weir JP (2005). Quantifying test-retest reliability using the intraclass correlation coefficient and the SEM. J Strength Cond Res.

[33] Riener R, Rabuffetti M, Frigo C (2002). Stair ascent and descent at different inclinations. Gait Posture.

[34] Alpkaya AT, Yılmaz M, Şahin AM, Mihçin DŞ (2024). Investigation of stair ascending and descending activities on the lifespan of hip implants. Med Eng Phys.

[35] McGuirk TE, Perry ES, Sihanath WB, Riazati S, Patten C (2022). Feasibility of markerless motion capture for three-dimensional gait assessment in community settings. Front Hum Neurosci.

[36] Chen X, Davis J (2008). An occlusion metric for selecting robust camera configurations. Mach Vis Appl.

[37] Kadaba MP, Ramakrishnan HK, Wootten ME (1990). Measurement of lower extremity kinematics during level walking. J Orthop Res.

[38] Gallagher KM, VandenBussche J, Callaghan JP (2013). Gait adaptations to different paths of stair descent. Gait Posture.

[39] Chai Y, Chen J, Hou M (2023). Gait strategies for individuals with knee osteoarthritis when transitioning between floor and stair walking. Front Physiol.

[40] Lay AN, Hass CJ, Gregor RJ (2006). The effects of sloped surfaces on locomotion: a kinematic and kinetic analysis. J Biomech.

[41] McIntosh AS, Beatty KT, Dwan LN, Vickers DR (2006). Gait dynamics on an inclined walkway. J Biomech.

[42] Protopapadaki A, Drechsler WI, Cramp MC, Coutts FJ, Scott OM (2007). Hip, knee, ankle kinematics and kinetics during stair ascent and descent in healthy young individuals. Clin Biomech (Bristol).

[43] Silverman AK, Neptune RR, Sinitski EH, Wilken JM (2014). Whole-body angular momentum during stair ascent and descent. Gait Posture.

[44] Cho H, Arnold AJ, Cui C (2023). Risky behavior during stair descent for young adults: Differences in men versus women. PLoS ONE.

[45] Tinetti ME, Baker DI, McAvay G (1994). A multifactorial intervention to reduce the risk of falling among elderly people living in the community. N Engl J Med.

[46] Grimmer M, Zeiss J, Weigand F (2020). Lower limb joint biomechanics-based identification of gait transitions in between level walking and stair ambulation. PLoS One.

[47] Bonanno M, Calabrò RS (2023). Robot-aided motion analysis inneurorehabilitation: benefits and challenges. Diagnostics (Basel).

[48] Mihcin S, Sahin AM, Yilmaz M (2023). Database covering the prayer movements which were not available previously. Sci Data.

[49] Kato K, Yoshimi T, Tsuchimoto S (2021). Identification of care tasks for the use of wearable transfer support robots - an observational study at nursing facilities using robots on a daily basis. BMC Health Serv Res.

[50] Kato K, Yoshimi T, Aimoto K, Sato K, Itoh N, Kondo I (2022). A rise-assisting robot extends life space and improves facial expressions of nursing home residents. BMC Health Serv Res.

[51] Kato K, Hashimoto Y, Aimoto K (2024). Electrocardiogram and respiration recordings show a reduction in the physical burden on professional caregivers when performing care tasks with a transfer support robot. Assist Technol.

[52] Kanko RM, Laende E, Selbie WS, Deluzio KJ (2021). Inter-session repeatability of markerless motion capture gait kinematics. J Biomech.

[53] Keller VT, Outerleys JB, Kanko RM, Laende EK, Deluzio KJ (2022). Clothing condition does not affect meaningful clinical interpretation in markerless motion capture. J Biomech.

[54] Mihcin S, Ciklacandir S, Kocak M, Tosun A (2021). Wearable motion capture system evaluation for biomechanical studies for hip joints. J Biomech Eng.

