# Supplementary figures and images for: Validation of Gait Kinematics With Ramp and Stair Ascent and Descent Revealed by Markerless Motion Capture in Simulated Living Space: Test-Retest Reliability Study
Source: JMIR Rehabil Assist Technol. 2025 May 15;12:e66886. doi: 10.2196/66886 (PMC12097655; doi:10.2196/66886)

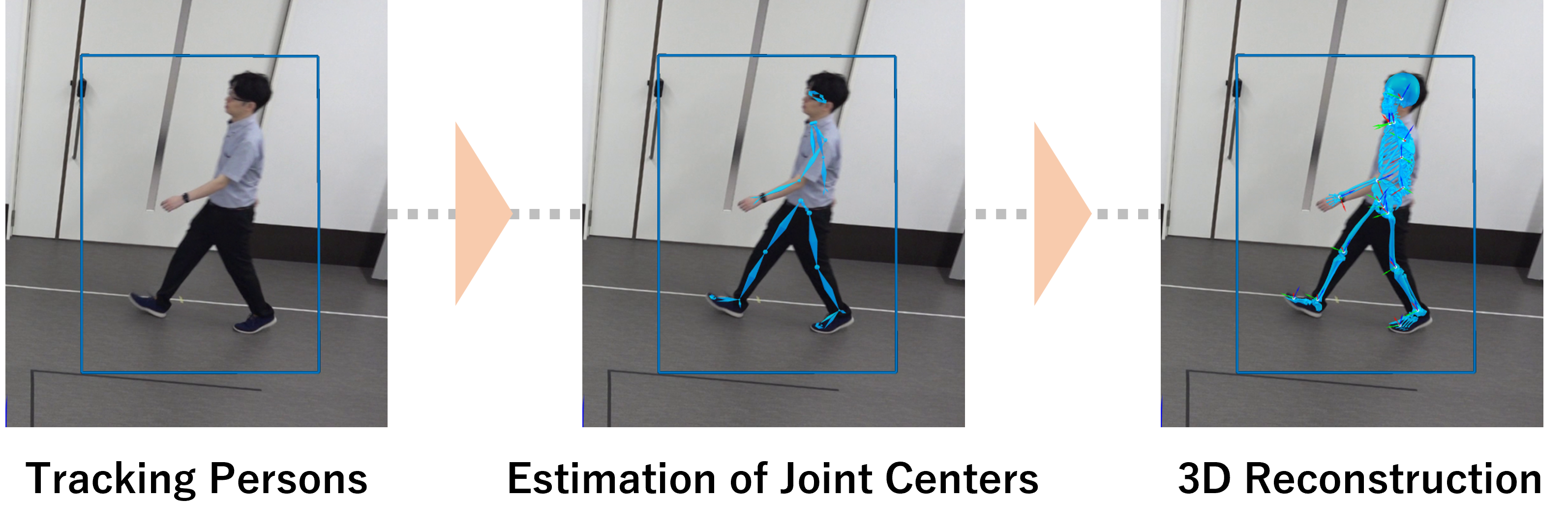

Supplement: Multimedia Appendix 1 [file rehab-v12-e66886-s001.png]
